# Prevalence and risk factors for hospital-acquired anemia in internal medicine patients: learning from the “less is more” perspective

**DOI:** 10.1007/s11739-022-03147-x

**Published:** 2022-11-08

**Authors:** Rosanna Villani, Antonino Davide Romano, Roberta Rinaldi, Moris Sangineto, Mariateresa Santoliquido, Tommaso Cassano, Gaetano Serviddio

**Affiliations:** 1 C.U.R.E. (University Centre for Liver Disease Research and Treatment), Liver Unit, Department of Medical and Surgical Sciences, University of Foggia, Viale Pinto 1, 71122 Foggia, Italy; 2 Department of Medical and Surgical Sciences, University of Foggia, Foggia, Italy

**Keywords:** Anemia, Hospital-acquired anemia, Iatrogenic anemia, Hemoglobin, Phlebotomy

## Abstract

Hospital-acquired anemia is defined as a new-onset anemia in hospitalized patients who have a normal hemoglobin level at admission. Its prevalence is unknown and most studies published on this topic have been conducted in intensive care unit patients with limited applicability to less acute settings, such as internal medicine wards. We conducted a retrospective study and enrolled 129 patients who were admitted to an Internal Medicine Unit between October 2021 and February 2022. The median value of phlebotomy during hospitalization was 46 ml (IQR 30–72 ml), whereas the median length of hospital stay was 9 days (IQR 5–13 days). The median value of hemoglobin reduction was −0.63 g/dl (*p* < 0.001) and the maximum value of drop in hemoglobin value was −2.6 g/dl. All patients who experienced a phlebotomy > 85 ml had a hemoglobin reduction > 0.6 g/dl. 20.9% of patients developed anemia during the hospital stay (7% moderate and 13.9% mild). No cases of severe anemia were observed. The volume of blood drawn during the hospital stay and the Hb value on admission were the only two variables statistically associated with the development of anemia, whereas gender, age, and chronic diseases, such as diabetes, history of cancer, or heart failure, were not. Strategies, such as elimination of unnecessary laboratory tests and the use of smaller tubes for blood collection, are needed to reduce the volume of iatrogenic blood loss and avoid blood wastage occurring during hospitalization in internal medicine patients.

## Introduction

Hospital-acquired anemia (HAA) is defined as a new-onset anemia in hospitalized patients who have a normal hemoglobin (Hb) level at admission [1]. Its prevalence is unknown, however it is estimated that 40%–74% of hospitalized patients develop a HAA before discharge [2–5] and that, in most cases, anemia is either moderate or severe [5].

Many potential etiologies may be involved in the development of HAA, such as shortened red blood cell (RBC) life span and decreased RBC production [1, 6]; however, the blood loss for diagnostic tests seems to play a pivotal role in the reduction of hemoglobin level [6].

During hospitalization, the amount of blood collected for diagnostic tests is between 8.5 and 12 times more than the instrument's analytic volume, and a median of about 2.0 ml is discarded for each tube [7]. Levi et al. estimated that annually 25 million L of blood drawn for diagnostic tests is thrown into waste containers in the Western countries [8].

Evidence suggests that patients who develop anemia during hospitalization are at high risk of adverse outcomes such as increased in-hospital mortality or longer hospital stays [5, 9] and that, moreover, the development of HAA is associated with increased hospital charges, ranging from + 6% for mild HAA to + 80% for severe HAA [5].

For all these reasons, HAA is currently considered a major clinical problem in hospitalized patients.

However, a large part of the current data on this topic have been conducted in intensive care unit patients and the results cannot be applied to more common settings with a lower intensity of medical care, such as internal medicine wards [3].

The few available and old studies, which have focused on internal medicine patients, had limited sample sizes and did not adequately control for the role of other potential causes of blood loss during hospitalization [10–13]. The most recent study addressing this topic in a general internal medicine setting was published in 2004 by Thavendiranathan et al*.* [3] who showed that, in a population of 404 patients, the volume of phlebotomy was a strong predictor of a drop in hemoglobin level and that, on average, every 100-mL phlebotomy was associated with a decrease in hemoglobin and hematocrit of 0.7 g/dL and 1.9%, respectively [3].

For all these reasons, HAA is a major challenge for the healthcare system to be addressed for the optimization of inpatient management particularly of “complex patients” admitted generally to internal medicine wards because of several and chronic-interrelated diseases; however, the magnitude, severity and impact on the clinical course of HAA in an internal medicine setting are largely unknown.

Therefore, our aim was to estimate the prevalence and severity of HAA and the impact of blood loss on changes in hemoglobin levels in hospitalized internal medicine patients.

## Materials and methods

### Study design and patient population

We conducted a retrospective study and analyzed data from clinical records of patients admitted to the Liver and Internal Medicine Unit of the University of Foggia between October 2021 and February 2022.

Patients who died during the hospital stay were excluded a priori from the study.

All medical records were reviewed by two investigators (R.R. and R.V.) to identify any exclusion and inclusion criteria.

All included patients were 18 years or older and had one hemoglobin value on admission and within 72 h before discharge.

The exclusion criteria were.

1. Presence of anemia on admission
2. Medical conditions that may cause anemia, such as gastrointestinal bleeding, hemolysis, hematuria, hemoptysis, hemothorax;
3. Hematological malignancies
4. Therapy that could affect hemoglobin levels, such as iron, chemotherapy, erythropoietin, or red blood cell transfusion;
5. Chronic kidney disease (stage 4–5) according to the KDIGO classification [14]).

Anemia was defined as Hb < 12 g/dl in women and < 13 g/dl in men.

Different degrees of anemia were defined according to the WHO guidelines [15] as follows:

- Mild anemia (10–12 g/L for women; 10–13 g/L for men)
- Moderate anemia (8–10 g/L for both sexes)
- Severe anemia (< 8 g/L for both sexes)

Demographics, such as age and gender, and laboratory data were recorded.

Finally, we recorded the parenteral fluid therapy to analyze the impact of plasma dilution on the final delta change in Hb values.

### Laboratory data

Hb, hematocrit, platelets, white blood cell count, alanine transaminases, aspartate transaminases, gammaglutamyltransferase (GGT), alkaline phosphatase (ALP), albumin, total bilirubin, serum creatinine, creatinine, blood iron level, ferritin and types of collection tubes used for each phlebotomy were recorded.

### Calculation of blood phlebotomy volume

Total phlebotomy volume per patient was calculated based on the number and type of blood tubes collected during hospitalization. Complete blood count was obtained using a 3 ml tube; electrolytes, renal and liver profiles were studied using 5 ml tubes, whereas 8 ml was the volume calculated per each bottle for blood cultures.

As previously reported [3], 15 random days during our study, we calculated for each type of specimen tube (except for bottles for blood cultures) the height to which each tube was filled. The mean of the height was used to obtain the volume of blood collected in each tube. In our study population, the mean fill volume was 68% with a standard deviation (SD) of 27%.

### Statistical analysis

Statistical analysis and graphs were performed using SPSS (Statistical Package for the Social Sciences, version 20, Armonk, New York, NY, USA) and GraphPad Prism, version 9 (La Jolla, CA, USA).

Quantitative variables are presented as median and interquartile ranges (IQR). Univariate and multivariate logistic regression analysis was used to identify the associations between clinical parameters and new-onset of anemia during the hospitalization. A *p* value of < 0.05 was considered as statistically significant.

## Results

### Study population

A total of 362 hospital admissions were screened. Of these, 233 patients (64.4%) were excluded according to the exclusion criteria, whereas the remaining 129 patients (35.6%) were included in the final analysis.

Complete characteristics of the study population at baseline are reported in Table 1. hsCRP values at baseline and discharge were 40.5 mg/dl (IQR 9.9–81.7) and 24.6 mg/dl (7.15–29.8) respectively.

**Table 1:** Baseline characteristic of study population

Variable	Study population, *N* = 129
Age, years	72 (59–82)
Male gender, %	64.3%
Length of hospitalization, days	9 (5–13)
Volume of phlebotomy, ml	46 (30–72)
Comorbidities	
Cancer	33.4%
COPD	27.9%
Heart failure	25.6%
Diabetes	30.2%
Total bilirubin, normal range: 0.20–1.20 mg/dl	0.81 (0.63–1.28)
Creatinine, normal range: 0.20–1.20 mg/dl	0.89 (0.69–1.19)
Urea, normal range: 10–50 mg/dl	43 (35–61)
Ferritin, normal range: 25–380 ng/dl	157 (71–251)
Iron level, normal range: 60–150 μg/dl	79 (37–118)
Hemoglobin value on admission, g/dl	13.2 (12.6–14.1)
MCV, normal range: 80–99 fl	87.3 (82.5–94.2)
RDW, normal range: 11.6–16.5%	14.1 (13.1–14.8)
Patients who developed anemia during the hospitalization	20.9%
Reduction of Hb value during hospitalization, g/dl	−063 (−0.95;0.01)
Admission diagnosis (1 or more)	
Dyspnea	26.1%
Abdominal or epigastric pain	19.6%
Chest pain	11.4%
Fever	10.6%
Vomit	8.1%
Acute liver hepatitis	6.5%
Decompensated heart disease	4.9%
Pneumonia	4.9%
Atrial fibrillation	4.1%
Disorders of consciousness (syncope/stupor/confusional state)	3.3%
Others^#^	20.3%

*Values are expressed as median and interquartile ranges; ^#^dizziness, pulmonary effusion, liver lesions of unknown origin, dysphagia, weight loss, cough, diarrhea, decompensated liver disease, skin complaints, thrombocytopenia.

### Change in hemoglobin levels during hospitalization

After comparison between Hb values recorded on admission versus values before discharge, 68.3% patients showed a negative balance in Hb level during hospitalization.

20.9% (*N* = 27) of included patients, who were not anemic on admission, became anemic during the hospitalization.

**Fig. 1:**
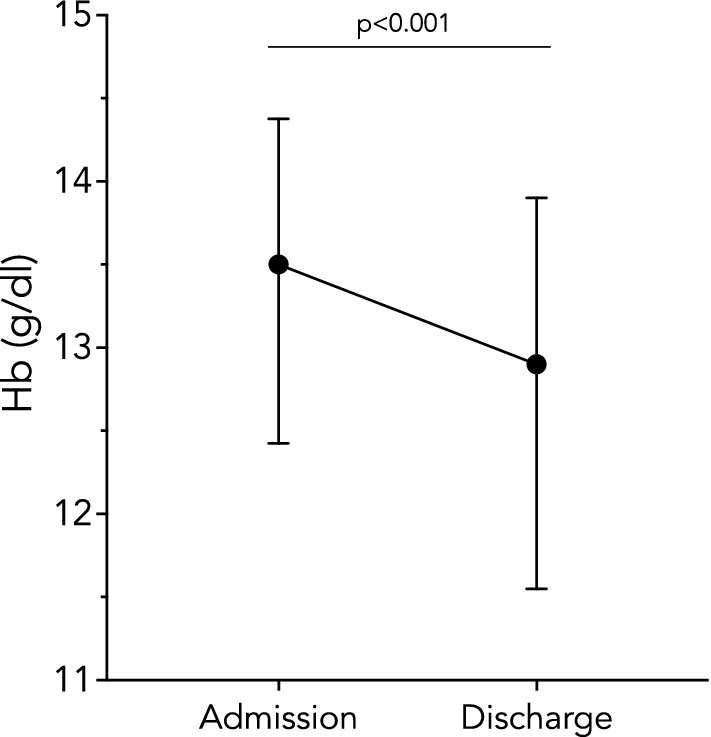
Hemoglobin levels at admission and discharge. Values are expressed as medians and interquartile ranges

The Hb drop during the hospital stay was statistically significant in our study population *p* < 0.001; Fig. 1). The maximum reduction in Hb value observed during the hospital stay was −2.3 g/dl, whereas the median value of reduction was −0.63 g/dl (IQR −1,1; 0) (*p* < 0.001).

**Fig. 2:**
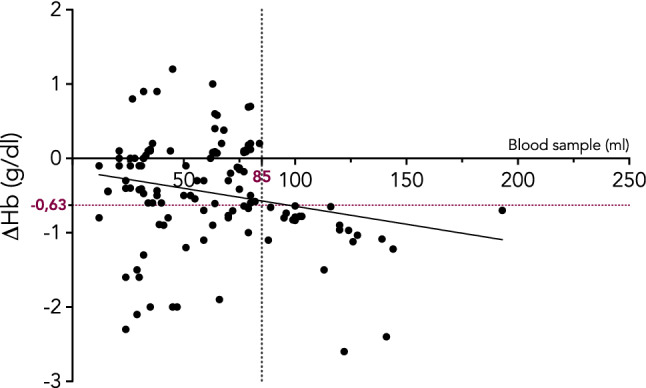
Correlation between the volume of hemoglobin drawn and hemoglobin reduction during the hospital stay. Above 85 ml (red vertical line), all patients experienced a negative delta change in hemoglobin value (> 0.63 g/dl)

Figure 2 shows the correlation between the volume of blood loss during the hospital stay and delta changes in Hb values for each patient.

In our study population, all patients who experienced a volume of blood drawn more than 85 ml showed a reduction in Hb greater than −0.63 g/dl.

All patients who became anemic during hospitalization experienced a mild (61.5%) or moderate anemia (38.5%). We reported no cases of severe anemia and none required red blood cell transfusion.

### Predictors in hemoglobin drop of hospitalization

Univariate and multivariate analyses (Table 2) were performed to investigate the risk factors involved in the development of anemia during hospitalization.

The volume of blood drawn during the hospital stay and the Hb value on admission (at baseline) are the only two variables, which were statistically significant, whereas gender, age, and chronic diseases, such as diabetes, history of cancer, or heart failure, were not significant.

The volume of phlebotomy was a statistically significant risk factor after adjusting for other factors.

Particularly, in our study population, for every milliliter of blood drawn, a drop in Hb value of 0.011 g/dl (IQR −0.003; −0.019) was observed.

**Table 2:** Risk factors for hemoglobin change during hospitalization

Variable	Univariate analysis			Multivariate analysis		
	*B*	OR (95% CI)	*p*-value	*B*	OR (95% CI)	*p*-value
Gender, female	0.239	1.271 (0.299–5.406)	0.746	−0.928	0.395 (0.049–3.194)	0.384
Age, years	0.021	1.021 (0.978–1.066)	0.338	0.022	1.022 (0.947–1.104)	0.573
Volume of blood drawn, mL	0.023	1.230 (1.040–1.402)	0.016	0.041	1.402 (1.030–1.803)	0.034
Length of hospitalization, days	0.036	1.047 (0.969–1.131)	0.246	−0.012	0.988 (0.851–1.147)	0.875
Hb level at admission, g/dl	−0.957	0.384 (0.217–0.679)	0.001	−1.283	0.277 (0.115–0.667)	0.004
hsCRP, mg/dl	−0.013	0.896 (0.886–1.016)	0.291	−0.025	0.884 (0.727–1.013)	0.263

### Parenteral fluid therapy during hospitalization

We analyzed fluid therapy given to 80 patients during the hospital stay. In this subgroup, only 4 patients received 24-h parenteral nutrition (infusion rate: 30 ml/h for the first 48 h then 60 ml/h). Nine patients received intravenous medications which did not require concurrent use of fluids and four patients did not receive any intravenous therapy.

Sixty-seven patients received fluid therapy during the hospitalization and the daily median value of fluid load was 400 cc (IQR 200–750 cc).

Except for patients with parenteral nutrition, all patients received the fluid therapy according to a thrice-daily administration regimen [infusion time ranges between 15 min and 3 h; median infusion rate 5 ml/min (IQR 3–9 ml/min); maximum infusion rate 16 ml/min].

## Discussion

Anemia is a condition commonly observed in hospitalized patients, which is generally multifactorial [16].

Strong evidence has shown that anemia plays an active role in changing outcomes in hospitalized patients because it is associated with higher mortality and increase in length of stay [17].

Irrespective of comorbidities and admission diagnosis, in hospitalized patients a common and particular type of anemia, the so-called hospital-acquired anemia, (HAA) may occur.

HAA is defined as the new-onset drop in hemoglobin value below the limit of normality in patients who have normal levels of hemoglobin before the admission [2].

Consequences associated with anemia remain incompletely defined and just like any other type of anemia, HAA seems to be associated with worse clinical outcomes.

Even if the etiology is complex, healthcare practice seems to increase the risk of developing HAA [1]. The incidence of HAA has not been well defined; however, available studies suggested that about 25% of patients have HAA and higher percentages (up to 75%) are reached when the nadir value of hemoglobin observed during the hospitalization is considered [5, 18, 19].

Most articles addressing the topic of new-onset anemia in hospitalized patients have been performed in critical ill populations admitted to intensive care units or patients hospitalized for acute myocardial infarction [16, 20].

In 2004, Thavendiranathan et al*.* studied 404 hospitalizations in general internal medicine and found that the mean volume of phlebotomy during the hospital stay was 74.6 ml (standard deviation 52.1 ml) [3] and that phlebotomy is a strong predictor of drop in hemoglobin and hematocrit after adjusting for potential predictors, such as length of hospital stay, admission hemoglobin/hematocrit value, age, Charlson comorbidity index, and admission intravascular volume status. In their study population, on average, every 100-mL phlebotomy was associated with a decrease in hemoglobin value of 0.7 g/dL.

However, the authors did not exclude from final analysis patients who had anemia on admission even if medical conditions causing anemia such as gastrointestinal bleeding were not included.

We included in our study only patients who had normal hemoglobin value on admission to address the real impact of the only hospitalization on the drop in the hemoglobin value.

The exclusion of patients with anemia or at high risk of anemia has significantly affected the number of patients included in the final analysis; however, this made it possible for us to study the true effect of hospitalization on hemoglobin levels because patients admitted to the internal medicine units have generally several comorbidities which may be involved in the development of anemia and be confounding factors when we aim at studying the pros and cons of healthcare practices on the patient’s health.

We found that one patient out of five became anemic during the hospitalization due to the healthcare practices. The volume of blood loss and the Hb level on admission were statistically significant and associated with the development of anemia. In our study population, a median decrease in Hb values of 0.63 g/dl was observed whereas all patients who experienced a blood loss > 85 ml had a decrease > 0.63 g/dl. Age, gender, length of stay were not risk factors for hemoglobin change during hospitalization.

We did not consider in our analysis patients with other potential sources for hemoglobin reduction; therefore, our data estimate the real impact of the only medical practices on the changes in hemoglobin level.

Salisbury et al. studied a large population (*N* = 2909) of patients admitted for acute myocardial infarction and found that about 45% developed anemia and 26% of them developed a moderate or severe anemia. In their cohort, patients who had mild anemia experienced a blood loss of 83.5 ml (SD 52.0 ml) whereas moderate or severe anemia was associated with a blood loss from phlebotomy of 173.8 ml (SD 139.3 ml) [18].

In our study population, we found that for every milliliter of blood drawn, a drop in Hb value of 0.011 g/dl was observed. This value was greater than values reported by Thavendiranathan et al., however, difference in sample size and inclusion criteria could have been involved in different final results.

Our findings showed that 20.9% of our patients developed anemia and one-thirds (7%) of them developed a moderate anemia, whereas two-thirds had mild anemia (13.9%).

Therefore, our results suggest a lower rate of anemia and a less severe HAA in general medical settings in comparison with data reported by other authors who addressed the incidence and the risk factors for anemia in critical ill populations. However, the question remains about the risk of moderate anemia, which is 7% in our population, but it could be a significant percentage from a large-scale perspective.

On admission at our hospital, patients are automatically subjected to blood draw for complete blood count and serologic tests; moreover, laboratory tests are often ordered daily even when no clinical changes occur and clinicians seem to have little interest in the large amount of blood wasted because of unnecessary laboratory tests [1].

Probably, defensive medicine may play a pivotal role because ordering blood tests driven by no clinical reasons cannot be otherwise justified [1, 21].

Strategies, such as elimination of unnecessary laboratory tests and the use of smaller tubes for blood collection, could be effective in reducing the volume of iatrogenic blood loss and avoiding blood wastage with no negative impact on the course of management and availability of tests supporting the clinical team.

As concerns the relationship between parenteral fluid load during hospitalization and plasma dilution, Hahn et al. have previously demonstrated that the fluid loading phase may associate with plasma dilution ranging between 2 and 25% [22].

An infusion rate of at least 50 ml/min, over at least 40 min is able to determine a plasma dilution of 20% which corresponds to an increase of blood volume of 10%. However, at the end of this loading fluid phase, additional 25 ml/min is required to maintain dilution over time. When loading fluid phase is not followed by further fluid infusion, restoration of plasma volume occurs within about 120 min because urinary excretion is proportional to plasma dilution. Specific nomograms are available to calculate the plasma dilution according to the infusion time and infusion rate [22]. After analysis of parenteral fluid given to our study population, we found that the fluid infusion rate was no higher than 16 ml/min and that, therefore, the expected plasma dilution ranged between 2 and 5% which corresponded to an increase in the blood volume of approximatively 1–2.5%.

These data support the absence of significant impact of fluid therapy on Hb values in our study population which included carefully selected patients.

Indeed, the iatrogenic hemodilution also called “dilutional anemia” has been widely reported in literature [23–25], but it has been more often observed in critical ill patients who require the admission to the intensive care units and the administration of large volume of intravenous fluid such as 3 L within 24 h or 5 L within 72 h [24].

Our study has several limitations: the small number of patients enrolled, the retrospective design and the lack of data from patients with concurrent chronic diseases which may have a significant impact on the hemoglobin level per sè, such as end-stage kidney failure or hematological diseases. Moreover, we intentionally excluded patients with anemia at hospital admission to investigate only the potential role of the medical care practices in the development of anemia; therefore, future studies are needed to address the impact of hospitalization on the temporal trend of hemoglobin levels in patients with anemia on admission and the role of concurrent hematological disease in developing anemia during the hospital stay.

## Data Availability

All data generated or analysed during this study are included in this published article.

## Declarations

### Statement of human and animal right

All procedures performed in this study involving human participants were in accordance with the ethical standards of the institutional and/or national research committee and with the 1964 Helsinki declaration and its later amendments or comparable ethical standards.
